# Re-evaluating currently available data and suggestions for planning randomised controlled studies regarding the use of hydroxyethyl starch in critically ill patients - a multidisciplinary statement

**DOI:** 10.1186/cc12845

**Published:** 2013-07-26

**Authors:** Patrick Meybohm, Hugo Van Aken, Andrea De Gasperi, Stefan De Hert, Giorgio Della Rocca, Armand RJ Girbes, Hans Gombotz, Bertrand Guidet, Walter Hasibeder, Markus W Hollmann, Can Ince, Matthias Jacob, Peter Kranke, Sibylle Kozek-Langenecker, Stephan Alexander Loer, Claude Martin, Martin Siegemund, Christian Wunder, Kai Zacharowski

**Affiliations:** 1 Department of Anesthesiology, Intensive Care Medicine and Pain Therapy, University Hospital Frankfurt, Theodor-Stern-Kai 7, 60590 Frankfurt am Main, Germany; 2 Klinik für Anästhesiologie, Operative Intensivmedizin und Schmerztherapie, Universitätsklinikum Münster, Albert-Schweitzer-Campus 1, Gebäude A 1, 48149 Münster, Germany; 3 Dipartimento dei Trapiant, Azienda Ospedaliera 'Ospedale Niguarda Ca' Granda', Piazza dell'Ospedale Maggiore 3, 20162 Milan, Italy; 4 Department of Anesthesiology, Ghent University Hospital, De Pintelaan 185, 9000 Ghent, Belgium; 5 Department of Anesthesia and Intensive Care Medicine, Medical School of the University of Udine, University of Udine, via Palladio 8, 33100 Udine, Italy; 6 Department of Intensive Care, University Hospital VU Medical Centre, De Boelelaan 1117, 1081 HV Amsterdam, The Netherlands; 7 Abteilung für Anästhesiologie und operative Intensivmedizin, AKh Allgemeines Krankenhaus der Stadt Linz GmbH, Krankenhausstrasse 9, 4020 Linz, Austria; 8 Reanimation Medicale, Hospital Saint Antoine, 184 rue du Faubourg, Saint Antoine, 75012 Paris, France; 9 Anästhesie, Intensiv- und Palliativmedizin, Krankenhaus der Barmherzigen Schwestern Ried, Schloßberg 1, 4910 Ried im Innkreis, Austria; 10 Department of Anesthesiology, Academic Medical Center Amsterdam (AMC), Meibergdreef 9, 1105 AZ Amsterdam, The Netherlands; 11 Department of Intensive Care, Erasmus MC University Medical Centre Rotterdam, Doctor Molewaterplein 50-60, 3015 GE Rotterdam, The Netherlands; 12 Klinik für Anästhesiologie, Klinikum der Universität München, Nußbaumstraße 20, 80336 München, Germany; 13 Department of Anaesthesia and Critical Care, University Hospital Würzburg, Oberdürrbacher Straße 6, 97078 Würzburg, Germany; 14 Department of Anaesthesia and Intensive Care, Evangelisches Krankenhaus, Hans-Sachs-Gasse 12, 1180 Vienna, Austria; 15 Department of Anesthesiology, University Hospital VU Medical Center, De Boelelaan 1117, 1081 HZ Amsterdam, The Netherlands; 16 Intensive Care and Trauma Center, Marseilles School of Medicine, Nord University Hospital, Chemin des Bourrely, 13915 Marseilles, France; 17 Department of Anaesthesia, Intensive Care and Rescue Medicine, State Hospital Baden AG, Im Ergel 1, 5404 Baden, Switzerland

## Abstract

**Introduction:**

Hydroxyethyl starch (HES) is a commonly used colloid in critically ill patients. However, its safety has been questioned in recent studies and meta-analyses.

**Methods:**

We re-evaluated prospective randomised controlled trials (RCT) from four meta-analyses published in 2013 that compared the effect of HES with crystalloids in critically ill patients, focusing on the adherence to 'presumably correct indication'. Regarding the definition of 'presumably correct indication', studies were checked for the following six criteria (maximum six points): short time interval from shock to randomisation (<6 h), restricted use for initial volume resuscitation, use of any consistent algorithm for haemodynamic stabilisation, reproducible indicators of hypovolaemia, maximum dose of HES, and exclusion of patients with pre-existing renal failure or renal replacement therapy.

**Results:**

Duration of fluid administration ranged from 90 min up to a maximum of 90 days. Four studies considered follow-up until 90-day mortality, three studies 28-/30-day mortality, whereas four studies reported only early mortality. Included studies showed a large heterogeneity of the indication score ranging between 1 and 4 points with a median (25%; 75% quartile) of 4 (2; 4).

**Conclusions:**

The most important question, whether or not HES may be harmful when it is limited to immediate haemodynamic stabilisation, cannot be answered yet in the absence of any study sufficiently addressing this question. In order to overcome the limitations of most of the previous studies, we now suggest an algorithm emphasising the strict indication of HES. Additionally, we give a list of suggestions that should be adequately considered in any prospective RCT in the field of acute volume resuscitation in critically ill patients.

## Introduction

Several previous trials [1-3] question the safety of hydroxyethyl starch (HES) solutions compared to crystalloid solutions in critically ill patients, whereas other trials did not suggest any adverse effects [4-7]. These diverging results may be the consequence of multiple factors including different patient populations and types of HES (particularly in terms of molecular weight, substitution coefficient, raw material, and concentration [8,9]). However, we believe that one of the most important factors has not yet been explored in detail. In contrast to current practice and to package inserts, colloids should primarily be supposed to replace intravascular volume loss and depletion and should be administered under strict conditions.

In previous trials [1-3], however, fluid therapy, including administration of HES, was often neither standardised nor limited regarding dose, time frame or selection of patients at risk. It is even more inscrutable as those studies recommending not using colloids in general on the intensive care unit (ICU) had stabilised their patients with colloids before randomisation [1-3].

One of the next fundamental problems in the field of fluid therapy in critically ill patients is the divergence and impreciseness of terms specifying fluid therapy at all during the last decades. In this respect, we choose to use the term 'fluid administration' for maintenance of fluid balance (a condition where crystalloids should be preferred) and the term 'acute volume resuscitation' for the treatment of acute volume depletion (a condition where colloids might be preferred).

The European Medicines Agency's (EMA's) Pharmacovigilance Risk Assessment Committee (PRAC) concluded on 14 June 2013, that the available evidence suggests that the benefits of solutions containing HES no longer outweigh their risks and therefore recommended that the marketing authorisations for these medicines be suspended. Unfortunately, the PRAC has extrapolated these results to all patients irrespective of underlying conditions although there are still ongoing controversies on this subject due to unpublished data (CRYSTAL [10], BaSES [7], RAFTinG [11] and so on), and the use of HES in non-septic patients (for example intraoperative use) has not been addressed in the aforementioned studies, raising concerns about the safety of HES.

In this review, we discuss recent prospective randomised controlled trials (RCTs) comparing HES with crystalloids for fluid therapy in critically ill patients focusing on the adherence to a 'presumably correct indication', and give suggestions for the design of future prospective RCTs.

## Material and methods

In a first step, we screened the four recent meta-analyses published in 2013 [12-15] including RCTs with critically ill patients following sepsis, trauma, burns or any other disease hospitalised in an ICU. The intervention group received any type of HES. Control patients received 0.9% saline, Ringer's acetate, or Ringer's lactate. We excluded trials that exclusively compared HES with either other synthetic colloid or albumin that may potentially induce equally harmful effect and thereby mask any effects of HES. We prospectively decided to include only studies published in English.

In a second step, we analysed studies on the adherence to 'presumably correct indication' using both the four meta-analyses [12-15] and original published manuscripts. Then, we defined the following six criteria and generated a six-point score:

1. Did the authors randomise patients within a maximum of 6 h after the first sign of shock? This arbitrary time period was chosen based on Rivers *et al. *[16] and the Surviving Sepsis Campaign Guidelines [17].

2. Did the authors restrict HES for initial volume resuscitation? (We acknowledge that this issue is in conflict with its licensing and marketing authorisation.)

3. Did the authors use any consistent algorithm for haemodynamic stabilisation?

4. Do baseline data enable identification of reproducible indicators of hypovolaemia, haemodynamic instability or increased lactate?

5. Did the authors adhere to the summary of product characteristics, particularly to maximal dose of HES per day (for example, 6% HES 130/0.4 <50 mL/kg; 6% HES 200/0.5 <33 mL/kg; 10% HES 200/0.5 <20 mL/kg)? (Details of product characteristics have recently been summarised [9]).

6. Did the authors adhere to safety issues of product characteristics, particularly to the contraindication for patients with pre-existing renal failure or renal replacement therapy (RRT)?

## Results and discussion

Detailed descriptions of the 11 included studies [1-7,18-21] are given in Table S1 in Additional file 1.

Four studies considered follow-up until 90-day mortality [1-3,18], three studies 28-/30-day mortality [6,7,19], whereas four studies reported only early mortality (24-h, ICU, or hospital mortality) [4,5,20,21].

The type of HES studied was 6% Voluven 130/0.40 (Fresenius Kabi, Bad Homburg, Germany) [2,4-7,18], 6% Elo-Haes 200/0.6 (Fresenius Kabi, Bad Homburg, Germany) [21], 6% Tetraspan 130/0.42 (B. Braun Melsungen, Melsungen, Germany) [3], 6% Hemohes 200/0.45 to 0.55 (B. Braun Melsungen, Melsungen, Germany) [20], 10% Hemohes 200/0.45 to 0.55 (B. Braun Melsungen, Melsungen, Germany) [1], and 10% pentastarch 200 to 300/0.5 [19].

Duration of fluid administration ranged from 90 min [6], 24 h [5,21], 96 h [1,18] up to a maximum of 90 days [2,3]. The ratio of the amounts of HES and crystalloids ranged between 1:1 [3,4,6,19,21], 1:1.1 [7], 1:1.2 [2,18,20], 1:1.3 [1], up to a maximum of 1:2.4 [5].

Results from analysing the likelihood of the adherence to a 'presumably correct indication' are summarised in Table 1 and are shown in detail in Table S2 in Additional file 2. Studies showed a large variability of the score ranging between 1 and 4 points with a median (25%; 75% quartile) of 4 (2; 4).

**Table 1:** Probability of 'presumably correct indication' (six-point score)

				Criteria for 'presumably correct indication'				
	Patients (total, n)	Score (Y, n)	Time interval (start <6 h)	Restricted to acute volume resuscitation (Duration)	Algorithm for fluid administration	Haemodynamic instability at randomisation	Maximum dose	Exclusion of renal failure/ RRT
Du, 2011 [4]	42	1	No	No	Y	No	No	No
Perner, 2012 (6S) [3]	804	1	No	No	No	No	Y	No^b^
Brunkhorst, 2008 (VISEP) [1]^a^	537	2	No	No	Y	No	No^c^	Y^d^
Myburgh, 2012 (CHEST) [2]	6,742	2	No	No	No	No	Y	Y
van der Heijden, 2009 [20]^a^	48	3	No	Y	Y	No	Y	n.a.
Dubin, 2010 [5]	20	4	Y	No	Y	Y	Y^e^	n.a.
Guidet, 2012 (CRYSTMAS) [18]	174	4	Y	No	Y	Y^f^	Y	No^g^
James, 2011 (FIRST) [6]	109	4	Y	No	Y	Y	No	Y
McIntyre, 2008 (FINESS) [19]^a^	40	4	Y	No	Y	Y	No^h^	Y^i^
Siegemund, 2013 (BaSES) [7]	241	4	Y	No	Y	n.a.	Y	Y
Vlachou, 2010 [21]^a^	26	4	Y	No	Y	No	Y	Y

^a^Molecular weight was 200 kDa, which is known as an independent risk factor for mortality; ^b^acute kidney injury, defined as renal SOFA score of ≥2, creatinine >1.9 mg/dL or urine output<500 mL/d, was present in up to 36% of patients; ^c^inconsistencies between study protocol specifications and published baseline data in up to 38% of patients; ^d^renal dysfunction (urine output ≤0.5 mL/kg/h for 1 h and/or serum creatinine >2 times normal ranges) was present in up to 10.9% of patients; ^e^data for body weight are not provided, ^f^personal communication; ^g^inconsistencies between study protocol specifications and published baseline data in up to 68% of patients; ^h^after 12 h, the quantity and type of fluid administered were at the discretion of the treating physician; ^i^limited to chronic renal failure with renal replacement therapy. Y = Yes; n.a.= not assessed; RRT, renal replacement therapy.

We address a highly controversially discussed issue - whether or not HES might be safe in specified subgroups of patients if correct indication is considered?

Four recent systematic reviews [12-15] have been published within the last couple of months focusing on HES and fluid therapy in critically ill patients. The main findings of these meta-analyses were that patients assigned to HES may have a statistically significant increased risk of mortality and increased risk of getting RRT. In detail, HES resulted in a significantly increased risk of mortality and receiving RRT in the 6S trial [3], a significantly increased risk of renal failure in the VISEP trial [1], whereas risk for mortality and renal failure did not differ in the CHEST trial [2] referring to the adjusted analysis published in the supplement, respectively.

Interestingly, these studies have, however, been extensively criticised on the basis of late enrolment of patients, inadequate evidence of hypovolaemia and the need for volume resuscitation, as well as the lack of properly targeted endpoints for resuscitation [22-24]. More importantly, almost all of the previous meta-analyses [12-15] analysed methodological quality criteria and risk of bias, but none of these reviews considered the large heterogeneity regarding clinical conditions and the flaws in study design of the included trials.

We believe that the design of these trials underestimated the importance of having haemodynamic endpoints and neglected the understanding of how fluids should be administered. Their conclusions that HES should be avoided will probably lead to inappropriate administration of large amounts of crystalloids, albumin and/or red blood cells in the future.

From a physiological point of view, acute volume resuscitation with colloids should result in less amounts of fluids needed for haemodynamic stabilisation compared to crystalloids [25]. Supporting this hypothesis, several studies showed a ratio of the amounts of HES and crystalloids that was higher than 1:1 [1,2,5,18,20]. In the 6S trial [3] contrarily, the cumulative amount of study drug did not differ between the HES and crystalloid group during the first 3 days. Also, the daily amount of study drug did not decrease over time in this trial, although the amount of fluids needed for acute volume resuscitation is theoretically supposed to decrease day by day, as septic patients can typically be stabilised within 48 to 72 h. Taken together, this strategy of fluid therapy may be associated with an increased risk of fluid overloading that may have contributed to the observed higher rate of patients getting RRT and mortality, at least in part [3].

Moreover, banishing colloids from clinical routine and preferentially using non-colloids might likely result in higher amounts of crystalloids, albumin and red blood cells needed to reach haemodynamic endpoints. This new paradigm might open the 'floodgates' for the use of even more amounts of crystalloid fluids potentially increasing the incidence of fluid overload and interstitial oedema. Recent observational studies [26-28] suggested that positive fluid balance is associated with increased morbidity and mortality. Obviously sicker patients who never reverse their capillary permeability deficit have a positive fluid balance leading to an increased risk of mortality. Nevertheless, using multivariate analysis, positive fluid balance remained an independent risk factor for 28-day mortality after adjustment for age, gender, diabetes, hypertension, diuretic use, non-renal sequential organ failure assessment (SOFA) and sepsis [26]. That is why the results of these studies should be critically re-evaluated, and why any planned prospective RCTs in the field of volume resuscitation in critically ill patient should adequately consider the suggestions outlined in this paper.

### General limitations of the included trials

We are concerned that major limitations may have biased previous study results, and that they are not adequately considered within the ongoing discussion. In this respect, we would like to highlight a few very important issues:

1. *Fluid therapy before inclusion of patients*

Up to 52% [3] and 59% [1] of all study patients had already received large amounts of colloids for acute haemodynamic stabilisation before formal randomisation. In this respect, a substantiate characterisation of HES-specific effects is very difficult, and late randomisation of patients may lead to inclusion of patients who have well exceeded predefined haemodynamic targets. It makes the whole story even more critical as those studies recommending not using colloids in general in the ICU had stabilised their patients with colloids initially before randomisation [1-3].

2. *Prolonged administration of HES*

In some studies, HES was allowed to be administered up to a maximum of 90 days [6,7]. However, prolonged administration of HES after the initial phase of haemodynamic instability increases the risk of giving HES to non-hypovolaemic patients [5-7,13,17]. Thus, persistent use with presumably weak indication has no beneficial effect or may even be harmful.

3. *No control of other risk factors for mortality/renal failure (for example, blood transfusion)*

More patients in the starch groups received packed red cells, although the transfusion algorithm was not standardised. A high priority should be assigned to this task, as transfusion of red cells is independently associated with higher mortality among patients in the intensive care unit.

4. *No protocol for renal replacement therapy (RRT)*

None of the trials used any protocol for RRT. However, this is of great importance, since mode, timing, dose and other device settings may have an impact on its efficacy and patient outcome [29-31]. As long as 'need for RRT' is a study endpoint, indication for RRT should clearly be specified.

5. *Study protocol violations*

Although exclusion criteria and maximum dose of HES were specified in the study protocols, conflicts between study protocol specifications and published baseline data were found in a few studies [1,3,18].

### Future perspectives - our 'eight suggestions'

HES is a potent drug with presumably beneficial characteristics, but also known dose-dependent adverse effects. Importantly, most of the early goal-directed approaches suggesting improved outcome after major surgery were based on colloid application [32-34]. To ensure a presumably positive benefit/risk ratio, indication of HES should be strictly limited to the restoration of intravascular volume in hypovolaemic patients (acute volume resuscitation). Otherwise, infusion of HES and any other fluid would result in hypervolaemia, thereby shedding the endothelial glycocalyx and aggravating the capillary leak syndrome [35], especially in septic patients. In this respect, randomisation of patients up to 24 h after diagnosis of severe sepsis may lead to the inclusion of patients who have exceeded predefined haemodynamic targets, since in the starch groups of the VISEP [1] and 6S trials [3], the median values were: central venous oxygen saturation, 75% and 75%; and serum lactate level, 2.2 and 2.0 mmol/L, respectively. This might explain why 411 out of the 798 patients included in the 6S trial (52%), and 315 out of the 537 patients included in the VISEP trial (59%), had already received colloids before randomisation, irrespective of group assignment and the risk of renal failure.

In accordance to the current surviving sepsis campaign bundles, initiation of acute volume resuscitation and vasopressor therapy should be completed within 3 to 6 h [17].

1. We suggest that initiation of HES infusion should be limited to a short time frame after onset of shock if acute volume resuscitation has started (for example, within a maximum of, in general, 6 h). (We acknowledge that no evidence-based clinical data exists that supports the arbitrary selection of this time period.)

Duration of fluid administration ranged from 90 min [6], 24 h [5,21], 96 h [1,18], up to a maximum of 90 days [2,3]. Only one trial exclusively restricted the use of HES to initial volume resuscitation for acute haemodynamic stabilisation [20]. In this respect, no conclusion as to the type of intravenous fluid to use during this sensitive period can be reached yet. Prolonged administration of HES after the initial phase of haemodynamic instability and longer than 24 h may even be associated with an increased risk of administrating HES to non-hypovolaemic patients, as this has been observed in several recent pragmatic trials [1-3,18,20]. These trials clearly indicate that persistent use with presumably weak indication has no beneficial effect or may even be harmful for critically ill patients.

2. We suggest that HES should be limited to acute volume resuscitation for initial haemodynamic stabilisation when hypovolaemia is present. We also suggest re-assessment of volume status with clearly defined stopping rules for HES. Total time period of acute volume resuscitation with HES should not last longer than 24 h (if volume responsiveness is still present beyond 24 h, further application of HES is not recommended). (We acknowledge that this suggestion is in marked contrast to current practice and in conflict with its licensing or marketing authorisation.)

In the two large pragmatic 6S and CHEST trials [2,3], patients were resuscitated according to the fluid algorithm of the participating ICU and the clinicians were allowed to vary the algorithm from patient to patient (for example sometimes the physiological variable could be a central venous pressure below 10 mmHg, which clearly has been shown to be unrelated to hypovolaemia [36]; in another patient this could be a heart rate above 90 beats per minute (bpm)). Although the use of varying algorithms between participating centres in pragmatic trials may increase the generalisability of the results, patients are at an increased risk of receiving overdosed HES in the absence of a standardised and reliable algorithm for volume resuscitation. Indeed, the lack of goal-directed fluid management may have caused overinfusion of HES, aggravated haemodilution and potentially increased risk of blood transfusions in the 6S [3], VISEP [1] and CHEST [2] trials, at least in part.

**3. We suggest using standardised and reliable algorithms of fluid responsiveness and predefined haemodynamic endpoints for acute volume resuscitation in order to restrict HES to hypovolaemic patients and to avoid hypervolaemia and any overdosing**.

As HES should be limited to hypovolaemic patients, physicians have to pay more attention at haemodynamic parameters and specific triggers for volume therapy before HES infusion starts. However, in the majority of studies [1-4,7,18,20,21], we could not fully reproduce adequate indicators for haemodynamic instability, hypovolaemia, nor increased lactate from published baseline data, respectively. Only three trials [5,6,19] reported data on clinical signs that reasonably indicate hypovolaemia, thereby demonstrating that criteria for 'presumably correct indication' were met.

4. We suggest that initiation of HES infusion should be limited to patients with haemodynamic instability primarily due to absolute or relative hypovolaemia. (We acknowledge that this suggestion is in contrast to current practice and in conflict with its licensing or marketing authorisation.)

All drug trials need to adhere to the summary of product characteristics in order to get approval from the authorities. In so far, almost every study considered the maximum dose in its study protocol apart from two studies [4,6]. However, it has to be acknowledged that the amounts of study drug reported did not (at least in some trials) include fluids/ colloids given in the operating room (for example in [2]). Nevertheless, we found two other studies [1,19] where in a relevant group of patients maximum daily dose was exceeded. One study did not provide data on maximal dose [5].

**5. We suggest that the maximum dose of HES should be complied with**.

Hyperoncotic starch solutions with a molecular weight of 200 kDa are known to result in an increased risk of renal failure with the need for RRT [1]. Now, the 6S trial alerts us that even HES solutions with a molecular weight of 130 kDa may lead to an increased risk of renal failure and/or need for RRT, when HES was presumably permanently administered in probably 'non-hypovolaemic' patients [3]. Contrarily, HES resulted in fewer patients with 'risk' and 'injury' according to the risk, injury, failure, loss, and end-stage renal disease (RIFLE) criteria in the CHEST trial, although a slightly higher (non-significant) number of patients received RRT [2]. Also, the CRYSTMAS study did not find any difference in RIFLE and acute kidney injury (AKI) criteria [18].

Considering the increased risk of renal failure, exclusion of patients with pre-existing renal failure (oliguria/anuria) and/or pre-existing RRT is another important issue regarding adherence to safety issues of product characteristics. It has to be highlighted, that most of the study protocols emphasised the exclusion of patients with pre-existing renal failure and/or RRT prior to randomisation. Under these conditions, good consistency between study protocol and baseline data was found in several studies [2,6,7,19,21]. However, we also found moderate implicit inconsistencies between study protocol specifications and published baseline data. Kidney dysfunction does not necessarily imply renal failure but rather impaired kidney function. In this respect, 'acute kidney injury' (defined as renal SOFA score of ≥2 or urine output <500 mL/d) was present in up to 36% of patients in the 6S trial [3], 'renal impairment' (defined as serum creatinine >3.39 mg/dL) was present in up to 68% of patients in the CRYSTMAS trial [18], and 'renal dysfunction' (defined as urine output ≤0.5 mL/kg/h for 1 h and/or serum creatinine >2 times normal ranges) was present in up to 11% of patients in the VISEP trial [1], respectively.

As the term 'renal failure' represents the end-stage of loss of kidney function, the concept of AKI creates a new paradigm to encompass the entire spectrum of the syndrome from minor changes in markers of renal dysfunction to requirement for RRT [37]. AKI is defined as any of the following: increase in serum creatinine by x0.3 mg/dL within 48 h; or increase in serum creatinine to 1.5 times baseline; or urine output <0.5 mL/kg/h for 6 h [38]. The current KDIGO Clinical Practice Guideline for Acute Kidney Injury [38] even suggests using isotonic crystalloids rather than colloids (albumin or starches) as initial management for expansion of intravascular volume in patients at risk for AKI or with AKI (recommendation level 2, grade B).

On the other hand, acute oliguria (urine output <0.5 mL/kg/h for a couple of hours) may be a strong indicator for acute hypovolaemia, a condition where acute volume resuscitation with colloids might be indicated.

Taking into account the current guidelines [38] and the increasing risk of kidney injury after prolonged administration of HES, we suggest an expansion of the exclusion criteria not only to renal failure (as this has been considered in previous trials in the past) but rather to AKI in the future.

6. We suggest the following three-step approach:

1. **If there is any evidence of pre-existing renal failure or even AKI, HES should not be given**.

2. **If there is no evidence of pre-existing renal failure or AKI but acute oliguria is present, HES might be indicated in combination with crystalloids, as oliguria could simply be the consequence of acute hypovolaemia**.

3. **If oliguria is unresponsive to acute volume resuscitation using HES within a time period of maximum 6 h, HES should be stopped**.

The next fundamental problem in the interpretation of the endpoints of previous studies, in particular need for RRT, is that standardised definitions for starting RRT have not yet been used in any of these studies, as no consensus exists so far. Further, RRT is not a single homogeneous therapy but rather there are diverse modes of therapy and various ways of providing RRT that might affect its efficacy and patient outcome [29-31]. These aspects refers to the mode (intermittent vs. continuous), timing of RRT initiation and discontinuation, dose, and practice variations (type of dilution, type of anticoagulation, and so on).

In this respect, we believe, as far as 'need for RRT' is used as a relevant study endpoint, a standardised study protocol clarifying mode, dose, type of anticoagulation and rules for starting and stopping of RRT are mandatory. This may be particularly of interest in multicenter trials were indication for RRT may differ between centres.

**7. We suggest that indication of RRT should be defined within a study protocol in advance in future studies, when RTT is used as a relevant study endpoint**.

The final aspect that we would like to address is quality of data documentation and providing raw data for meta-analyses. This issue primarily refers to large clinical trials whose results and conclusions have a major impact on medical practice, but also on the registration authority in the EMA or the Food and Drug Administration (FDA). In smaller studies, standardisation of any study protocol and adequate data collection is often simple, but sample size is also insufficient to power for relevant clinical endpoints. By contrast, large pragmatic trials with several hundreds or thousands of patients are sufficiently powered for 90-day mortality, but are highly sophisticated in terms of valid data collection. This all leads to the double-edged sword of conducting large well-powered clinical trials. In this respect, the 6S trial [3] is limited as data of circulatory parameters (for example, central venous oxygen saturation) were not registered in up to 67% of patients during the first 24 h, and also 3-day detailed data on fluid therapy are missing in up to 19% of patients, respectively.

In terms of raw data, arguments for making raw data more widely available and sharing data were once more repeated in a recent editorial in the *British Medical Journal *[39] to ensure independent scrutiny, testing of secondary hypotheses, aiding design of new studies, and simplifying data acquisition for meta-analyses.

**8. We suggest that the best quality of data documentation and adequate follow-up are mandatory in future trials. Making raw data freely available would enable both clinicians and scientists to understand and to interpret study data more appropriately**.

### Future perspectives - algorithm for clinical management and safety checklist

The most important question, whether or not HES may be harmful when it is limited to immediate haemodynamic stabilisation cannot be answered yet. Currently, no study is available that sufficiently addresses this question. However, the recent large-scale trials have nicely demonstrated that chronic use without proper indication and assessment may cause harm. This has been the strength of these trials and can be viewed as an important contribution to safety in the care of critically ill patients.

**Figure 1:**
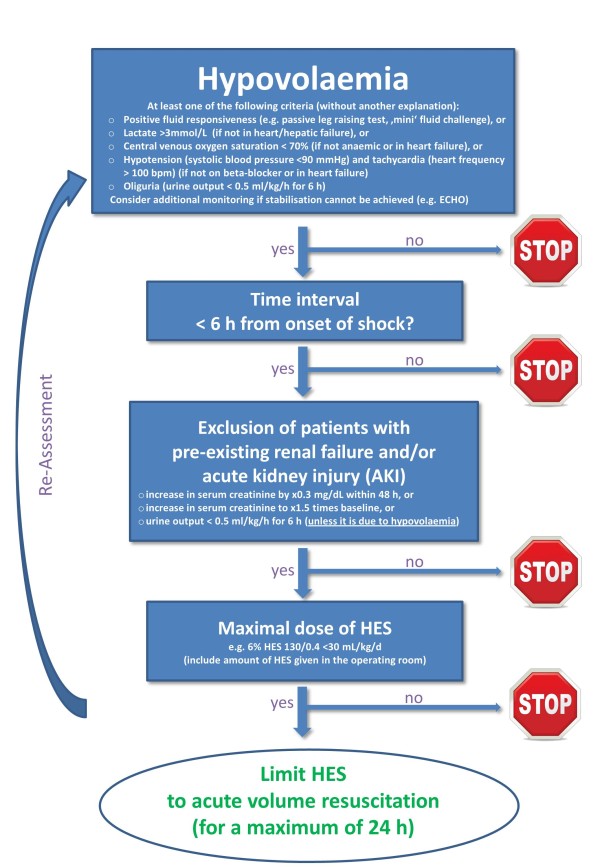
**Algorithm for clinical management considering strict indication**.

In fact, we need additional studies focusing on acute volume resuscitation and the initial phase of haemodynamic stabilisation. To overcome the above mentioned limitations of most of the previous trials, first, we suggest an algorithm for clinical management emphasising the strict indication of HES (Figure 1). This algorithm starts with the strict identification of patients with hypovolaemia. From our point of view, relevant hypovolaemia is likely if at least one of the following criteria is present: positive fluid responsiveness, lactate level >3 mmol/L, central venous oxygen saturation <70%, hypotension/tachycardia, and/or oliguria. As a second step, acute volume resuscitation with HES should be limited to a time interval of less than, in general, 6 h after onset of shock, to patients without pre-existing renal failure or AKI (unless oliguria is due to hypovolaemia), and to the initial phase of volume resuscitation (that should not last longer than 24 h). We acknowledge that the time interval of 6 h for starting HES might be tricky if no therapeutic intervention at all has been done, or if there are 'ups and downs' in the clinical course with a re-occurence of hypovolaemia. To keep it simple with a clear message and to avoid any prolonged administration of HES day by day, however, we suggest that use of HES should be restricted to the initial phase of volume resuscitation with a maximum time interval of 24 h.

**Figure 2:**
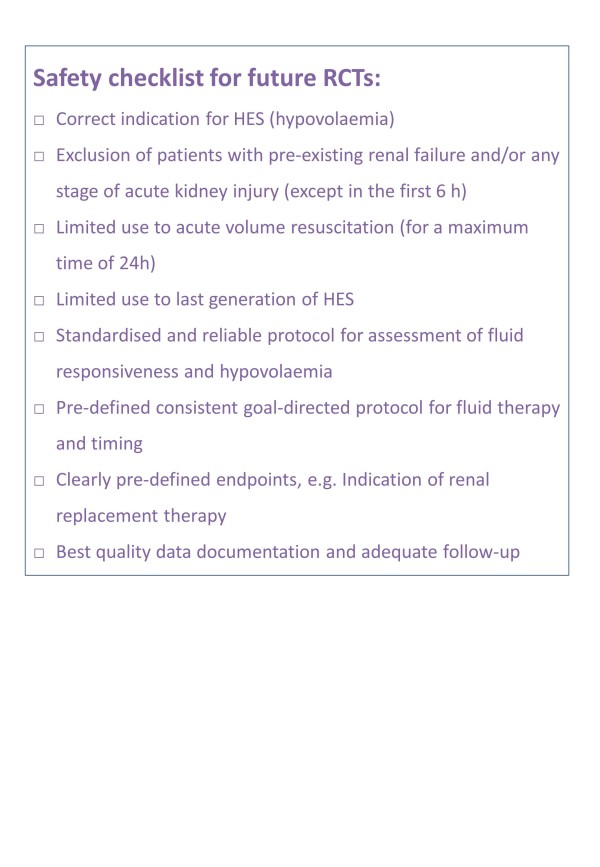
**Safety checklist for future prospective randomised controlled trials**.

As a final step, we give a list of suggestions that should be adequately considered by any planned prospective RCT in the field of acute volume resuscitation in critically ill patients in the future (Figure 2).

## Conclusions

As recent RCTs show large heterogeneity in terms of 'probably correct indication', the most important question, whether or not HES may be harmful when it is limited to acute volume resuscitation cannot be answered yet. We suggest an algorithm emphasising the strict indication of HES for patients with hypovolaemia limited to the initial phase of volume resuscitation. Additionally, we suggest a safety checklist for future prospective RCTs.

## Key messages

• The safety of HES has been questioned in recent trials, although full adherence to 'presumably correct indication', defined by short time interval from shock to randomisation, restricted use for initial volume resuscitation, use of any consistent algorithm for haemodynamic stabilisation, reproducible indicators of hypovolaemia, maximum dose of HES, and exclusion of patients with pre-existing renal failure or RRT, could not be found in any of these trials.

• The question, whether or not HES may be harmful when it is limited to immediate haemodynamic stabilisation, cannot be answered yet.

• We suggest an algorithm for clinical management emphasising the strict indication of HES.

• Further, we suggest a safety checklist for future prospective randomised controlled trials that might be important in the field of acute volume resuscitation in critically ill patients.

• The PRAC recommendation is viewed with concern, since it extrapolates not only from long-term use in septic patients to acute haemodynamic stabilisation in this cohort of patients but also to all licensed and not-licensed (off-label) use of HES.

## Abbreviations

AKI: acute kidney injury: HES, hydroxyethyl starch: ICU, intensive care unit: PRAC, Pharmacovigilance Risk Assessment Committee: RCT, randomised controlled trial: RIFLE: risk, injury, failure, loss, and end-stage renal disease: RRT, renal replacement therapy: SOFA, sequential organ failure assessment.

## Competing interests

PM received lectures fees from Pulsion Medical Systems and independent research grants from B. Braun Melsungen, Fresenius Kabi, Vifor Pharma, and CSL Behring. HVA received honoraria and travel expenses from Vifor Pharma, Abbott, and Fresenius Kabi. ADG received lecture/consultancy fees from Fresenius Kabi, CSL Behring and Grifols. HG held lectures for Fresenius Kabi, CSL Behring and Vifor Pharma.BG held lectures for Fresenius Kabi. He received independent research grants from Fresenius Kabi. He is member of the Grifols Albumin Advisory Board. MWH received lecture/consultancy fees and research grant support from B. Braun Melsungen, Fresenius Kabi, and CSL Behring. CI has received honoraria and independent research grants from Fresenius Kabi, Baxter Healthcare, and B. Braun Melsungen. MJ has held lectures for Fresenius Kabi, Baxter, B. Braun, Serumwerk Bernburg and CSL Behring. He received independent research grants from Serumwerk Bernburg, CSL Behring and Fresenius Kabi. He is member of the Grifols Albumin Advisory Board. PK received lecture fees from Fresenius Kabi and a research grant as the lead investigator (LKP) for the planning of a clinical trial investigating the initial haemodynamic stabilisation in severe sepsis. SK received honoraria for lectures, travel reimbursement and grants from B. Braun, Fresenius Kabi and CSL Behring. MS held lectures for Fresenius Kabi, B. Braun and and CSL Behring. CW received honoraria and independent research grants from Fresenius Kabi. KDZ received consultancies, honoraria and grants from Fresenius Kabi, B. Braun, and Vifor Pharma. All other authors declare that they have no competing interests with regard to this topic.

## Authors' contributions

PM and KZ drafted the manuscript. HVA, ADG, SDH, GDR, ARG, HG, BG, WH, MWH, CI, MJ, PK, SK, SAL, CM, MS and CW conceived of the study, participated in its design and coordination, and helped to draft the manuscript. All authors read and approved the final manuscript.

## Supplementary Material

Additional File 1 **Characteristics of studies (alphabetical order)**. Click here for file

Additional File 2 **Probability of 'presumably correct indication' (alphabetical order)**. Click here for file
