# Supplementary material for: Re-evaluating currently available data and suggestions for planning randomised controlled studies regarding the use of hydroxyethyl starch in critically ill patients - a multidisciplinary statement
Source: Crit Care. 2013 Jul 26;17(4):R166. doi: 10.1186/cc12845 (PMC4056523; doi:10.1186/cc12845)
Supplement: Additional File 1 — Characteristics of studies (alphabetical order). [file cc12845-S1.DOCX]

**Table S1. Characteristics of studies (alphabetical order)**

| **Study** | **Study design** | **Population** | **Study drug (type of HES)** | **Inclusion criteria** | **Exclusion criteria** | **Severity of illness** | **Cumulative amount colloid vs. crystalloid (ratio)** | **Mortality** |
| --- | --- | --- | --- | --- | --- | --- | --- | --- |
| Brunkhorst, 2008[1] | RCT multi-center, two-by-two factorial trial, double-blind, in 18 centers from Germany | 537 pts with severe sepsis or septic shock.  N=262 10% HES 200/0.45-0.55,  N=275 Ringer’s lactate.  In Ringer’s lactate group, 58% received colloids in the 12h prior randomisation (725 (500-1000mL)); 26.6% received “non-study colloids” (albumin, dextran, gelatin and HES) during study. | 10% Hemohes 200/0.45-0.55 (B. Braun Melsungen AG, Melsungen, Germany) | Severe sepsis or septic shock; eligible if the onset of the syndrome was <24 h before admission to the ICU or <12 h after admission if the condition developed in the ICU. | >1000 mL HES within 24 h before study inclusion, pre-existing renal failure requiring dialysis or a creatinine level ≥ 320 μmol/l (3.6 mg/dL), known allergy against HES, intracerebral haemorrhage, heart failure, immunosuppression, high dosage of steroids or AIDS, moribund due to coexisting disease, order to withhold or withdraw therapy. | APACHE II: HES 20.1 (6.7), control 20.3 (6.7) | 4 days:  cumulative dose 70.4 (33.4- 144.2) mL/kg  Ratio 1: 1.32 | 28-day,  90-day |
| Du, 2011[2] | RCT single-center, un-blind, in China | 41 pts with severe acute pancreatitis  N=20 6%HES 130/0.4,  N=21 Ringer’s lactate for 8 days | 6% Voluven 130/0.40 (Fresenius Kabi, Bad Homburg, Germany); waxy-maize | Age 18-70 years, confirmed diagnosis of severe acute pancreatitis, inclusion within 72 h after onset of symptoms. | History of allergy to HES, history of cardiac dysfunction, history of renal insufficiency, malignancy, or immunodeficiency, history of other colloid intravascular volume-replacement regimens within 24 h before enrollment, serum albumin level < 25 g/L, coagulopathy, possible death within 48 h after enrollment. | APACHE II: HES 12 (4), control 11 (4) | First 24 h:  5,300 (1,250) mL vs. 5,300 (1,250) mL  8 days:  30 L vs. 30 L (recalculated from Figure)  Ratio 1:1 | Hospital |
| Dubin, 2010[3] | RCT multi-center, single-blind, 2 center in Argentina | 20 pts with severe sepsis  N=9 6%HES 130/0.4,  N=11 0.9%saline | 6% Voluven 130/0.40 (Fresenius Kabi, Bad Homburg, Germany); waxy-maize | Confirmed or suspected infection plus 2 or more signs of SIRS and tissue hypoperfusion (MAP <65 mmHg despite a crystalloid fluid challenge of 20 mL/kg or lactate ≥4 mmol/L). | Stroke, acute coronary syndrome, status asthmaticus, cardiac arrhythmias (as a main diagnosis), active gastrointestinal haemorrhage, seizures, burns, trauma, need of immediate surgery, terminal cancer, immunosuppression, no resuscitation order, delayed admission to the ICU from the emergency department (>4 h), or previous resuscitation > 1500 mL of fluids. | SOFA:  HES 8.1 (2.5); saline 8.9 (3.6) | First 24 h:  2,610 (885) mL vs. 6,254 (2,603) mL  Ratio 1:2.4 | 24-hour |
| Guidet, 2012[4] | RCT multi-center, double-blind, in 24 centers from France and Germany | 196 pts. with severe sepsis,  N=100 6%HES 130/0.4,  N=96 0.9%saline  Efficacy analysis included n=174 who reached haemodynamic stability (HES: n=88, saline: n=86). | 6% Voluven 130/0.40 (Fresenius Kabi, Bad Homburg, Germany); waxy-maize | Severe sepsis with hypovolaemia | Related to pre-existing renal impairment (creatinine >3.39 mg/dL, anuria lasting >8 h despite fluid resuscitation, RRT), related to the potential effect on the primary endpoint (volume expansion with >3 L of fluid (crystalloid and/or colloid) since diagnosis of severe sepsis or refractory septic shock, norepinephrine or epinephrine at a dose >0.5 μg/kg/min | Mean SOFA: HES 7.9, control 9.1; mean SAPS II: HES 50, control 53 | Until stabilisation: 1,379 (886) mL vs.1,709 (1,164) mL  Ratio 1:1.24  4 days: cumulative 2,615 (1,499) mL vs. 2,788 (1,799) mL  Ratio 1:1.06 | 28-day,  90-day |
| James, 2011[5] | RCT single-center, double-blind, South Africa | 109 pts with trauma;  N= 56 6%HES 130/0.4,  N= 53 0.9% saline | 6% Voluven 130/0.40 (Fresenius Kabi, Bad Homburg, Germany); waxy-maize | Penetrating or blunt trauma;  requiring >3L volume resuscitation; aged 18–60 years | Fluid overload pulmonary oedema, pre-existing renal failure with oliguria or anuria; dialysis treatment before the injury; severe head injury/intracranial bleeding; >6 h after injury; any colloid before randomisation | SOFA: HES 6 (0–19), control 4 (0–11) in blunt trauma | First 24 h:  Penetrating 5,093 (2,733) mL vs. 7,473 (4,321) mL  Ratio 1:1.47  Blunt 6,113 (1,919) vs. 6,295 (2,197) mL  Ratio 1:1 | 30-day |
| McIntyre, 2008[6] | RCT multi-center, double-blind, 3 centers in Canada, 1 center in New Zealand | 40 pts with septic shock  N= 21 10%pentastarch 200-300/0.5,  N=19 0.9%saline  Prior randomisation, up to 500mL colloid were allowed in both groups. | 10% pentastarch 200-300/0.5 | Early septic shock with 3 criteria: 1) hypotension, 2) at least 2 SIRS criteria, 3) a suspected or confirmed infectious source. | > 500 mL of colloid (5% albumin or pentastarch) or 2000 mL of crystalloid fluid, other forms of shock (haemorrhagic, cardiogenic or obstructive shock), acute myocardial infarction, von Willebrand's disease, previous severe reaction to HES, chronic renal failure requiring dialysis, immediate need for surgery, projected life expectancy <3 months | APACHE II: pentastarch 21.1 (6.1); saline 20.2 (6.3) | First 12 h: 5,200 (1,900) mL vs. 5,100 (2,100)  Ratio 1:1 | 28-day |
| Myburg, 2012[7] | RCT multi-center, double-blind, in 32 centers from Australia and New Zealand | 7,000 pts with ICU admission (28.8% sepsis; 7.9% trauma)  N=3,315 6%HES 130/0.4,  N=3,336 0.9%saline  In saline group,15 % of pts. received up to 1000mL HES in the 24 h prior randomisation | 6% Voluven 130/0.40 (Fresenius Kabi, Bad Homburg, Germany); waxy-maize | All ICU pts. needing fluid resuscitation, as judged by the ICU clinicians and supported by at least 1 objective physiological criterion (HR >90 beats/min, SBP < 100 mmHg or MAP <75 mmHg or at least  40 mmHg decrease in SBP or MAP from the baseline, CVP <10 mmHg, pulmonary artery wedge pressure <12 mmHg, respiratory variation in systolic or MAP >5 mmHg, capillary refill time >1 second, U/O <0.5 mL/kg/h). | RRT(or RRT within the next 6 h), known allergic reaction to HES, intracranial haemorrhage, creatinine >350 μmol/L and U/O ≤10 mL/h over 12 h, hypernatremia (sodium >160 mmol/l), hyperchloremia (chloride >130 mmol/l), >1000 mL HES within the 24 h before randomisation, cardiac surgery, burns, liver  transplantation surgery, life expectancy < 90 days, limitation of therapy order. | APACHE II: HES 17 (12-22), control 17 (12-23) | first 4 days: 526 (425) mL vs. 616 (488) mL daily average  Ratio 1:1.17 | 90-day |
| Perner, 2012[8] | RCT multi-center, double-blind, in 26 centers from Denmark, Norway, Finland, and Iceland | 798 pts with severe sepsis,  N=398 6%HES 130/0.42,  N=400 Ringer’s acetate  In Ringer’s acetate group, 42 % of pts. received colloids in the 24h prior randomisation (500 (500-1000) mL) | 6% Tetraspan 130/0.42 (B. Braun Melsungen, Melsungen, Germany);  potato starch | Pts who fulfilled criteria for severe sepsis within the previous 24 h and need fluid resuscitation in the ICU, as judged by the ICU clinicians | RRT, kidney or liver transplantation, burn injury >10% of body surface, intracranial bleeding, serum potassium >6 mmol/L within 6 h before screening, >1000 mL of synthetic colloid before randomisation | SOFA: HES 7 (5-9), control 7 (5-9); SAPS II: HES 50 (40-60), control 51 (39-62) | First 3 days: 3,000 (1,507- 5,100) mL vs. 3,000 ( 2,000 -5,750) mL  Ratio 1:1 | 90-day |
| Siegemund, 2013[9]# | RCT single-center, double-blind; in Switzerland (Basel) | 241 pts with sepsis, severe sepsis, septic shock;  N=117 6%HES 130/0.40;  N=124 0.9%saline | 6% Voluven 130/0.40 (Fresenius Kabi, Bad Homburg, Germany); waxy-maize | Suspected or proven infection and 2 of the following 6 criteria: temperature <36 or >38.3°C, HR> 90 beats/min, tachypnea > 20/min or a arterial pCO_2_ < 4.25 kPa, white blood cell count > 12000 or < 4000, SBP <90 mmHg or MAP < 65 mmHg, altered mental state or oliguria | Allergy against HES, chronic renal insufficiency with haemodialysis, acute kidney injury (creatinine >350 μmol/l, colloid application before study inclusion | SOFA: HES 7 (4-11), control 8 (4-11);  SAPS: HES 46 (32-61), control 47 (29-60) | 5 days:  3,775 (2,018-6,347) mL  vs.  4,125 (2,500-6,730) mL  Ratio 1:1.09 | 30-day |
| Van der Heijden, 2009[10] | RCT single-center, single-blind, in The Netherlands | 48 pts with hypovolaemia (n=24 sepsis, n=24 surgery)  N=12 0.9%saline,  N=12 4%gelatin,  N=12 6%HES 200/0.45-0.55,  N=12 5%albumin | 6% Hemohes 200/0.45-0.55 (B. Braun Melsungen AG) | Clinical hypovolaemia was defined by SBP ≤110 mmHg and CVP ≤12 mmHg at PEEP ≤15 cm H_2_O (CVP ≤16 mm Hg at PEEP >15 cm H_2_O) | Age >78 years, known anaphylactoid reaction to colloid fluids, life expectancy < 24 h. | APACHE II: Colloid 10 (4-23), control 10 (6-23) | 90 min:  1,500 (1,100-1,800) mL  vs.  1,800 (850–1,800) mL  Ratio 1:1.2 | ICU |
| Vlachou, 2010 [11] | RCT single-center, unblind, Great Britain | 26 pts with burns  N=14 Hartmann’s solution,  N=12 6%HES 200/0.6 supplemented one-third of the crystalloid-predicted requirement | 6% Elo-Haes 200/0.6 (Fresenius-Kabi, Bad Homburg, Germany) | All adult acute burns admissions to the University Hospital Birmingham Burns Centre between May 2004 and May 2006, with injury exceeding 15% total body surface area were considered for inclusion | Burn > 80% total body surface area, transfer delay > 6 h from the time of injury, renal impairment (creatinine >130 mmol/L), haematological evidence of a bleeding diathesis. | No score provided | First 24 h:  8,650 vs. 8,450 mL  Ratio 1:1 | Hospital |

Key characteristics of studies including design, sample size, inclusion/exclusion criteria, analysis method, and measured outcomes.

APACHE, Acute Physiology And Chronic Health Evaluation; CVP, central venous pressure; HR, heart rate; HES, hydroxyl-ethyl starch; ICU, intensive care unit; MAP, mean arterial pressure; n.a., not assessed; PEEP, positive end-expiratory pressure; pts, patients; RCT, randomised controlled trial; SAPS, Simplified Acute Physiology Score; RRT, renal replacement therapy; SBP, systolic blood pressure; ScvO_2_, central venous oxygen saturation; SIRS, systemic inflammatory response syndrome; SOFA, Sequential Organ Failure Assessment; U/O, urine output.

Data are provided as mean (SD) or median (25%-75% inter-quartile range) or otherwise specified.

# Study was extracted from the recent meta-analysis by Haase et al. [12], detail data were added by personal communication.

**References**

1. Brunkhorst FM, Engel C, Bloos F, Meier-Hellmann A, Ragaller M, Weiler N, Moerer O, Gruendling M, Oppert M, Grond S *et al*: **Intensive insulin therapy and pentastarch resuscitation in severe sepsis**. *N Engl J Med* 2008, **358**:125-139.

2. Du XJ, Hu WM, Xia Q, Huang ZW, Chen GY, Jin XD, Xue P, Lu HM, Ke NW, Zhang ZD *et al*: **Hydroxyethyl starch resuscitation reduces the risk of intra-abdominal hypertension in severe acute pancreatitis**. *Pancreas* 2011, **40**:1220-1225.

3. Dubin A, Pozo MO, Casabella CA, Murias G, Palizas F, Jr., Moseinco MC, Kanoore Edul VS, Palizas F, Estenssoro E, Ince C: **Comparison of 6% hydroxyethyl starch 130/0.4 and saline solution for resuscitation of the microcirculation during the early goal-directed therapy of septic patients**. *J Crit Care* 2010, **25**:659 e651-658.

4. Guidet B, Martinet O, Boulain T, Philippart F, Poussel JF, Maizel J, Forceville X, Feissel M, Hasselmann M, Heininger A *et al*: **Assessment of hemodynamic efficacy and safety of 6% hydroxyethylstarch 130/0.4 vs. 0.9% NaCl fluid replacement in patients with severe sepsis: The CRYSTMAS study**. *Crit Care* 2012, **16**:R94.

5. James MF, Michell WL, Joubert IA, Nicol AJ, Navsaria PH, Gillespie RS: **Resuscitation with hydroxyethyl starch improves renal function and lactate clearance in penetrating trauma in a randomized controlled study: the FIRST trial (Fluids in Resuscitation of Severe Trauma)**. *Br J Anaesth* 2011, **107**:693-702.

6. McIntyre LA, Fergusson D, Cook DJ, Rankin N, Dhingra V, Granton J, Magder S, Stiell I, Taljaard M, Hebert PC: **Fluid resuscitation in the management of early septic shock (FINESS): a randomized controlled feasibility trial**. *Can J Anaesth* 2008, **55**:819-826.

7. Myburgh JA, Finfer S, Bellomo R, Billot L, Cass A, Gattas D, Glass P, Lipman J, Liu B, McArthur C *et al*: **Hydroxyethyl starch or saline for fluid resuscitation in intensive care**. *N Engl J Med* 2012, **367**:1901-1911.

8. Perner A, Haase N, Guttormsen AB, Tenhunen J, Klemenzson G, Aneman A, Madsen KR, Moller MH, Elkjaer JM, Poulsen LM *et al*: **Hydroxyethyl starch 130/0.42 versus Ringer's acetate in severe sepsis**. *N Engl J Med* 2012, **367**:124-134.

9. Siegemund M: **BaSES Trial: Basel Starch Evaluation in Sepsis**. *Personal communication, ClinicalTrialsgov Identifier: NCT00273728* 2013.

10. van der Heijden M, Verheij J, van Nieuw Amerongen GP, Groeneveld AB: **Crystalloid or colloid fluid loading and pulmonary permeability, edema, and injury in septic and nonseptic critically ill patients with hypovolemia**. *Crit Care Med* 2009, **37**:1275-1281.

11. Vlachou E, Gosling P, Moiemen NS: **Hydroxyethylstarch supplementation in burn resuscitation--a prospective randomised controlled trial**. *Burns* 2010, **36**:984-991.

12. Haase N, Perner A, Hennings LI, Siegemund M, Lauridsen B, Wetterslev M, Wetterslev J: **Hydroxyethyl starch 130/0.38-0.45 versus crystalloid or albumin in patients with sepsis: systematic review with meta-analysis and trial sequential analysis**. *BMJ* 2013, **346**:f839.
