# Supplementary material for: Re-evaluating currently available data and suggestions for planning randomised controlled studies regarding the use of hydroxyethyl starch in critically ill patients - a multidisciplinary statement
Source: Crit Care. 2013 Jul 26;17(4):R166. doi: 10.1186/cc12845 (PMC4056523; doi:10.1186/cc12845)
Supplement: Additional File 2 — Probability of 'presumably correct indication' (alphabetical order). [file cc12845-S2.DOCX]

**Table S2. Probability of *‘presumably correct indication’* (alphabetical order)**

| **Study** | **Time interval / Duration** | | **Algorithm for fluid administration** | **Haemodynamic instability at randomisation** | **Maximum dose** | **Renal failure/ RRT at randomisation** |
| --- | --- | --- | --- | --- | --- | --- |
| Brunkhorst, 2008 (VISEP) | | Start: Inclusion of pts. was allowed up to 24 h after diagnosis of sepsis before ICU admission (or up to 12 h after ICU admission)  Duration: 96 h | During 96 h after randomisation, volume resuscitation was mandatory if CVP < 8 mmHg, MAP <70 mmHg or ScvO_2_<70%; the treating physician decided on further measures (fluid repletion, vasopressor and/or inotropic drugs) to raise MAP and ScvO_2_ into predefined ranges. | Haemodynamic instability/ increased lactate not reproducible:  MAP: HES 76 (67-85) mmHg, control 75 (68-85) mmHg; CVP: HES 12 (8-15) mmHg, control 12 (8-15) mmHg; ScvO_2_: HES 75 (69-81) %, control 74 (68-79)%; lactate: HES 2.2 (1.5-3.8) mmol/L, control 2.2 (1.5-4.3) mmol/L | Limit of 20 mL/kg/day; Dose limits were exceeded in >38 % of pts; cumulative dose 70.4 (33.4-144.2) mL/kg | Protocol: exclusion of pre-existing kidney failure requiring dialysis or creatinine > 320 mmol/l (3.6 mg/dL);  Baseline data: in 5.3 % (HES) and 10.9% (control) of pts. renal dysfunction (U/O  ≤0.5 mL/kg/h for 1 h despite adequate fluid loading and/or serum creatinine > 2x times normal ranges) |
| Du, 2011 | | Start: < 72 h after the onset of symptoms  Durations: 8 days | Fluid therapy targeting CVP > 8 mm Hg, U/O > 0.5 mL/kg/h, or a SBP > 90 mm Hg | Haemodynamic instability not reproducible:  SBP: HES 134 (13) mmHg, control 129 (12) mmHg; CVP: HES 9 (3) mmHg, control 9 (2) mmHg. | > 5,000mL/day, study medication was planned to be given for 8 days. | Baseline data: renal failure in 10% (HES) and 5% (control) of pts. |
| Dubin, 2010 | Start: < 4 h after ICU admission  Duration: 24 h | | Early goal directed therapy:  MAP > 65 mmHg, CVP 8-12 mm Hg, ScvO_2_ > 70 %; fluids if pts. responsive to crystalloid fluid challenges of 20 mL/kg or lactate > 4 mmol/L | Lactate: HES 3.0 (1.1) mmol/L, control 3.8 (2.3) mmol/L; pH HES 7.28 (0.10), control 7.23 (0.11); base excess HES -6.0 (4.4) mmol/L, control 9.1 (4.9) mmol/L | 2610 (885) mL/24h (data for body weight are not provided; assuming an average body weight of 75kg, mean dose was 34 mL/kg/d) | Not specified |
| Guidet, 2012 (CRYSTMAS) | | Start: initial stabilisation < 4 h;  Data: time to haemodynamic stability: HES 11.8 (10.1) h, saline 14.3 (11.1) h.  Duration: 96 h | Fluid therapy targeting MAP ≥ 65 mmHg and at least 2 of the following parameters maintained for 4 h: CVP 8-12 mmHg, U/O >2 mL/kg, or ScvO_2_ > 70%. | Fluid intake prior to randomisation: HES 35.5 (25.3) mL/kg, control 39.9 (28.6) mL/kg. Further haemodynamic data are not specified, but personal communication emphasised haemodynamic instability. | 50 mL/kg/day on the first day, 25 mL/kg/day on the second to fourth days, according to patient needs; cumulative dose over 4 days 2,615 (1,499) mL. | Protocol: exclusion of serum creatinine >3.39 mg/dl, or RRT;    Baseline data: renal impairment prior randomisation (serum creatinine >3.39 mg/dL) in 64% (HES) and 68% (control) of pts. |
| James, 2011 | Start: <6h after injury  Duration: up to 30 d | | Study algorithm for the administration of fluids and assessment of resuscitation including MAP, CVP, ScvO_2_, U/O | Protocol: SBP <100mmHg, or clinical indications of shock: <estimated blood loss replaced; HR>110 beats/min; poor peripheral perfusion; poor saturation signal; cold peripheries; metabolic acidosis: pH <7.25  Data: lactate >4 mmol/L in all groups | >5000mL at first day  (Body weight was 72.2 (7.6) and 76.8 (14.4) kg;  resulting in >60mL/kg/d ) | Exclusion: renal failure with oliguria or anuria; or dialysis treatment before the injury |
| McIntyre, 2008 (FINESS) | Start: Time to inclusion: HES 1.2 (0.9- 2.3) h, control 1.5 (1.0-3.0) h.  Duration: 12 h (after 12 h, the quantity and type of fluid administered was at the discretion of the treating physician) | | Early-goal directed therapy: 500 mL boluses of study fluid to a maximum of 28 mL/kg (or 3 L within 12 h) according to protocol, aiming at CVP 8-12 mmHg; then open-label saline, later target normal MAP, ScvO_2_ >70% | Protocol: hypotension defined by any of the following: (i) SBP < 90 mmHg or < 40 mmHg below baseline; or (ii) MAP < 65 mmHg; or (iii) need for a vasopressor; or (iv) need for further fluid resuscitation as determined by the treating physician after receiving at least 1 L of crystalloid fluid within the first 8h of the first hypotensive event. | Patients received a maximum of 28 mL/kg (or 3000 mL) of study fluid during the 12 h study period. However, after 12 h, the quantity and type of fluid administered was at the discretion of the treating physician. | Exclusion: chronic renal failure |
| Myburg, 2012 (CHEST) | | Start/ Duration: At any time during the patient’s ICU admission (until ICU discharge, death, or 90 days after randomisation)  Time from ICU admission to randomisation: HES 11 (157) h, control 11 (165) h | Need for fluid judged by the ICU clinicians and supported by at least 1 criterion (HR >90 beats/min, SBP < 100 mmHg or MAP <75 mmHg, CVP <10 mmHg, pulmonary artery wedge pressure <12 mmHg, respiratory variation in systolic or MAP >5 mmHg, capillary refill time >1 second, U/O <0.5 mL/kg for 1 h). | Haemodynamic instability/ increased lactate not reproducible:  MAP: HES 74 (15) mmHg, control 74 (15) mmHg; CVP: HES 10 (5) mmHg, control 9 (5) mmHg; lactate: HES 2.1 (2.0) mmol/L, control 2.0 (1.5) mmol/L | 50 mL/kg/day | Exclusion: RRT(or RRT within the next 6 h); serum creatinine ≥ 350μmol/L and U/O ≤ 10mL/h over 12 h. |
| Perner, 2012 (6S) | | Start: Inclusion of pts. was allowed up to 24 h after fulfilling criteria of severe sepsis  Duration: 90 d | Trial fluid was used when ICU clinicians judged that volume expansion was needed in the ICU for a maximum of 90 days | Haemodynamic instability/ increased lactate not reproducible:  CVP: HES 10 (7-13) mmHg, control 10 (8-13) mmHg; ScvO_2_: HES 75 (67-83) %, control 73 (65-82)%; lactate: HES 2.0 (1.3-3.5) mmol/L, control 2.1 (1.4-3.7) mmol/L | 33 mL/kg/day of ideal body weight  42 % of pts. had received colloids in the 24h prior randomisation (700 (500-1000) mL) | Protocol: exclusion of any form of RRT;    Baseline data: acute kidney injury (defined as renal SOFA score of ≥2, creatinine >1.9 mg/dL or U/O <500 mL/d) in 36% (HES) and 35% (control) of pts. |
| Siegemund, 2013 (BaSES)# | Start: Immediately after diagnosis of severe sepsis or shock and ICU admission  Duration: 5 d | | Strict alternating application of 1000 mL study fluid (HES vs. saline) and 1000mL Ringer's lactate (first hour 1000 mL of each); algorithm targeting MAP > 65 mmHg, CVP 8-12 mmHg, ScvO_2_>70% | Protocol: MAP < 65 mmHg, HR >90 beats/min;  Data not specified | Max. dose 50 mL/kg/day; cumulative dose 3,775 (2,018-6,347) mL within the first 5 days | Exclusion: creatinine > 350 μmol/L; or chronic haemodialysis |
| van der Hejden, 2009 | | Start: < 3 h after surgery or gastrointestinal haemorrhage;  < 12 h after meeting criteria for sepsis  Duration: 90 min | Boluses of max. 200 mL/ 10 min (max. fluid challenge 1800 mL in 90 min). Clinical hypovolaemia was defined by SBP ≤110 mmHg and CVP ≤12 mmHg at PEEP ≤15 cm H_2_O (CVP ≤16 mmHg at PEEP >15 cm H_2_O) | Haemodynamic instability/ increased lactate not reproducible:  Intrathoracic blood volume index: colloid 1042 (476–1764) mL/m^2^, saline 897 (640–1510) mL/m^2^; cardiac index: colloid 3.4 (2.0–8.2) mL/min/m^2^, saline 3.9 (2.4–5.5) mL/min/m^2^ | Max. fluid challenge was 1800 mL in 90 min. | Not specified |
| Vlachou, 2010 | Start: 4(3–5) h post-injury  Duration: 24 h | | U/O: 0.5–1 mL/kg/h in uncomplicated burn injury and 1–2 mL/kg/h in the presence of inhalation injury; MAP >70 mmHg; HR <120 beats/min | Haemodynamic instability/ increased lactate not reproducible: median (95%CI) base excess: HES-supplemented -2.8 (-6 to -0.9) mmol/L, control -1.1 (-4.6 to 2.4) mmol/L; median (95%CI) MAP: HES-supplemented 82 (69–91) mmHg, control 86 (75–90) mmHg | 33 mL/kg/24 h; 1585mL/24 h | Exclusion: renal impairment (serum creatinine >130 mmol/L) |

CVP, central venous pressure; HES, hydroxyl-ethyl starch; HR, heart rate; ICU, intensive care unit; MAP, mean arterial pressure; pts, patients; PEEP, positive end-expiratory pressure; RRT, renal replacement therapy; SBP, systolic blood pressure; ScvO_2_, central venous oxygen saturation; SOFA, Sequential Organ Failure Assessment; U/O, urine output.

Data are provided as mean (SD) or median (25%-75% inter-quartile range) or otherwise specified.

# Study was extracted from the recent meta-analysis by Haase et al. [1], detail data were added by personal communication.

References:

1. Haase N, Perner A, Hennings LI, Siegemund M, Lauridsen B, Wetterslev M, Wetterslev J: **Hydroxyethyl starch 130/0.38-0.45 versus crystalloid or albumin in patients with sepsis: systematic review with meta-analysis and trial sequential analysis**. *BMJ* 2013, **346**:f839.
